# Decrypting phytomicrobiome of the neurotoxic actinorhizal species, *Coriaria myrtifolia*, and dispersal boundary of *Frankia* cluster 2 in soil outward compatible host rhizosphere

**DOI:** 10.3389/fmicb.2022.1027317

**Published:** 2022-11-10

**Authors:** Erik Swanson, Imed Sbissi, Amir Ktari, Hafsa Cherif-Silini, Faten Ghodhbane-Gtari, Louis S. Tisa, Maher Gtari

**Affiliations:** ^1^Department of Molecular, Cellular and Biomedical Sciences, University of New Hampshire, Durham, NH, United States; ^2^LR Ecologie Pastorale, Institut des Régions Arides, Médenine, Tunisia; ^3^USCR Bactériologie Moléculaire and Génomique, Institut National des Sciences Appliquées and de Technologie, Université de Carthage, Tunis Cedex, Tunisia; ^4^LR Microbiologie Appliquée, Département de Microbiologie, Faculté des Sciences Naturelles et de la Vie, Université Ferhat Abbas, Sétif, Algeria; ^5^Institut Supérieur de Biotechnologie de Sidi Thabet, Université de La Manouba, Biotechnopôle, Sidi Thabet, Sidi Thabet, Tunisia

**Keywords:** actinorhizal symbiosis, microbiome, endophyte, epiphyte, symbiont, *Coriaria myrtifolia*, 16S rRNA next-generation-sequencing-metabarcoding, *Frankia* cluster 2

## Abstract

The actinorhizal plant, *Coriaria myrtifolia,* is a neurotoxic plant species endemic to the western Mediterranean area, which forms a nitrogen-fixing symbiosis with members of *Frankia* cluster 2. Contrarily to other *Frankia* clusters, the occurrence and mode of dispersal for infective cluster 2 units outside of the host plant rhizosphere remains controversial. The present study was designed to investigate the structure of the microbiomes of *C. myrtifolia* phytosphere, rhizosphere, and soil samples extending outward linearly up to 1 km. Results showed that the epiphyte and endophyte communities were not significantly different from each other for most of the plant tissues. The communities associated with the below-ground tissues (nodule and root) were significantly different from those found on the above-ground tissues (fruit, leaves, and stems) and had a higher community richness. *Coriaria myrtifolia* phytomicrobiomes were dominated by *Cyanobacteria* for leaf, stem, and fruit while *Actinobacteria* and *Proteobacteria* were dominant in the root and nodule organelles. The nodule, a special niche for nitrogen fixation, was mainly inhabited by *Frankia* but contained several non-*Frankia* bacteria. Beside *Frankia* cluster 2, the presence of clusters 1, 4, and large numbers of cluster 3 strains have been detected in nodules, roots, and rhizospheres of *C. myrtifolia*. Despite *Frankia* being found in all plots using plant trapping bioassays with *C. myrtifolia* seedlings, *Frankia* cluster 2 was not detected in soil metagenomes showing the limits of detection by this approach. This result also suggests that in the absence of appropriate host plant species, *Frankia* cluster 2 has a reduced number of infective units present in the soil outward from the rhizosphere.

## Introduction

*Coriaria myrtifolia* is a shrub endemic to the Western Mediterranean found mainly in forests and scrublands (Gtari and Dawson, 2011; Ribeiro-Barros et al., 2019). All plant parts contain a sesquiterpenic lactone named coriamyrtine, which is responsible for poisoning of livestock and human (De Haro et al., 2005; Anadón et al., 2018). Neurotoxicity occurs upon ingestion of stems, branches, leaves, or fruits. Goats have the highest frequency of poisoning with lower rates in cattle and horses. The lag time for symptoms is short, 20 min to 2 h after ingestion of the plant (Faliu et al., 1985). Symptomatically, intoxication by the neurotoxin is characterized by ptyalism, mydriasis, sometimes chills, loss of appetite, and bloating. This event is followed by severe neurological signs including retching, trembling, violent seizures that last 10–15 min, muscular contractions, ataxia, anorexia, tachycardia, intense tachypnea and dyspnea, nystagmus, mydriasis, ptyalism, and bloating (Faliu et al., 1985; Anadón et al., 2018). If not treated, death occurs within 20 min to 2 h after ingestion. The use of *C. myrtifolia* in tanning and dyeing processes is well documented in medieval texts—mainly from Catalunya (Cardon and Pinto, 2007). The phytomicrobiome or plant microbiome consists of a diverse array of beneficial, neutral, or damaging microorganisms associated with all plant compartments; above (phyllosphere) or below (rhizosphere) ground as epiphytes (on surface), endophytes (within) or nearby plant tissues (Singh and Trivedi, 2017; Sharma et al., 2021). Phytomicrobiome has attracted extensive interest in recent years because of the crucial role it plays in plant growth, health, and ecological function and for current and future biotechnological applications (Turner et al., 2013). Our understanding of the phytomicrobiome has greatly increased using culture-independent approaches with high throughput technologies such as metabarcoding, metagenomics, metatranscriptomics, and metaproteomics. Nevertheless, phytomicrobiomes for native populations of trees and shrubs, especially in forests, have remained less characterized than for other herbaceous and cultivated plant species (Terhonen et al., 2019).

The ability of *C. myrtifolia* to bear nitrogen-fixing root nodules has been confirmed several times (Bond, 1962; Berg et al., 1999; Nouioui et al., 2014) and its microsymbiont was identified as a member of the genus *Frankia* (Becking, 1970), which forms a distinct lineage (Nick et al., 1992). *Frankia* found in other *Coriaria* species, *Datiscaceae*, *Dryadoideae*, and *Ceanothus* species (Nouioui et al., 2011, 2014) fall within cluster 2 of the four well-defined *Frankia* clusters (Normand et al., 1996; Ghodhbane-Gtari et al., 2010a). Members of *Frankia* cluster 2 are difficult to isolate in pure culture (Gtari et al., 2015). Although soil is considered a second ecological niche for *Frankia*, their free-living existence and dispersal mechanisms for their infective units have not been fully uncovered (Dawson, 2007). Currently, almost all information of the soil habitat was based on indirect approaches using either plant-trapping bioassays (Smolander and Sundman, 1987; Rönkkö et al., 1993; Burleigh and Dawson, 1994) or quantitative-PCR (Samant et al., 2012, 2016). The transient occurrence of the microsymbiont in host rhizosphere *via* released infective units from decaying nodules and long-distance dispersal outward dispersal from host rhizosphere is likely achieved through wind, water, and biological vectors (Dawson, 2007). Isolation of many *Frankia* strains in pure culture is strong indirect evidence of the ability of strains to thrive asymbiotically in soils. Infective units from clusters 1, 3 and 4 have been shown to be ubiquitously distributed in soils around the world, (Paschke and Dawson, 1992; Nalin et al., 1997; Gtari et al., 2004) even in newly deposited glacial till, volcanic lava deposits, and young sand dunes prior to colonization by host plants (Young et al., 1992). Moreover, *Frankia* strains from cluster 1 were found to occur abundantly in non-host rhizosphere (Smolander, 1990) and fresh lava deposit with the volcanophile actinorhizal plant species *Myrica* and *Casuarina* (Burleigh and Dawson, 1994; Yamanaka and Okabe, 2006; Kurten et al., 2008). For cluster 2, the situation remains more debatable (Battenberg et al., 2017; Ben Tekaya et al., 2017). It has been previously shown that strains from cluster 2 were able to persist in *Alnus glutinosa* rhizosphere for a decade in the absence of their compatible host plants (Nouioui et al., 2013). The time course of nodule development in the plant bioassay was relatively very long (18 months) and may be essential to increase *Frankia* cell units to reach a threshold allowing the initiation of root nodulation. This idea is coherent with claims that the absence of compatible host plant results in a rapid drop of infective units in soil outside of the compatible host rhizosphere (Benson and Silvester, 1993). The recent isolation of members of cluster 2 in axenic conditions provided evidence for saprophytic ability by some members of this group of *Frankia* strains (Gtari et al., 2015; Gueddou et al., 2019).

The objectives of the present study are to investigate (1) whether the wild status of the toxic actinorhizal species. *Coriaria myrtifolia,* might be able to shape the phylogenetic structures of its phytomicrobiome and (2) to deepen understanding of the spatial distribution of cluster 2 *Frankia* strains in the soil nearby (rhizosphere) and more distant from *C. myrtifolia* trees, as well as spatial patterns of associated soil microbiomes.

## Materials and methods

### Sampling, *Frankia* isolation, bioassay, and DNA extraction

**Figure 1:**
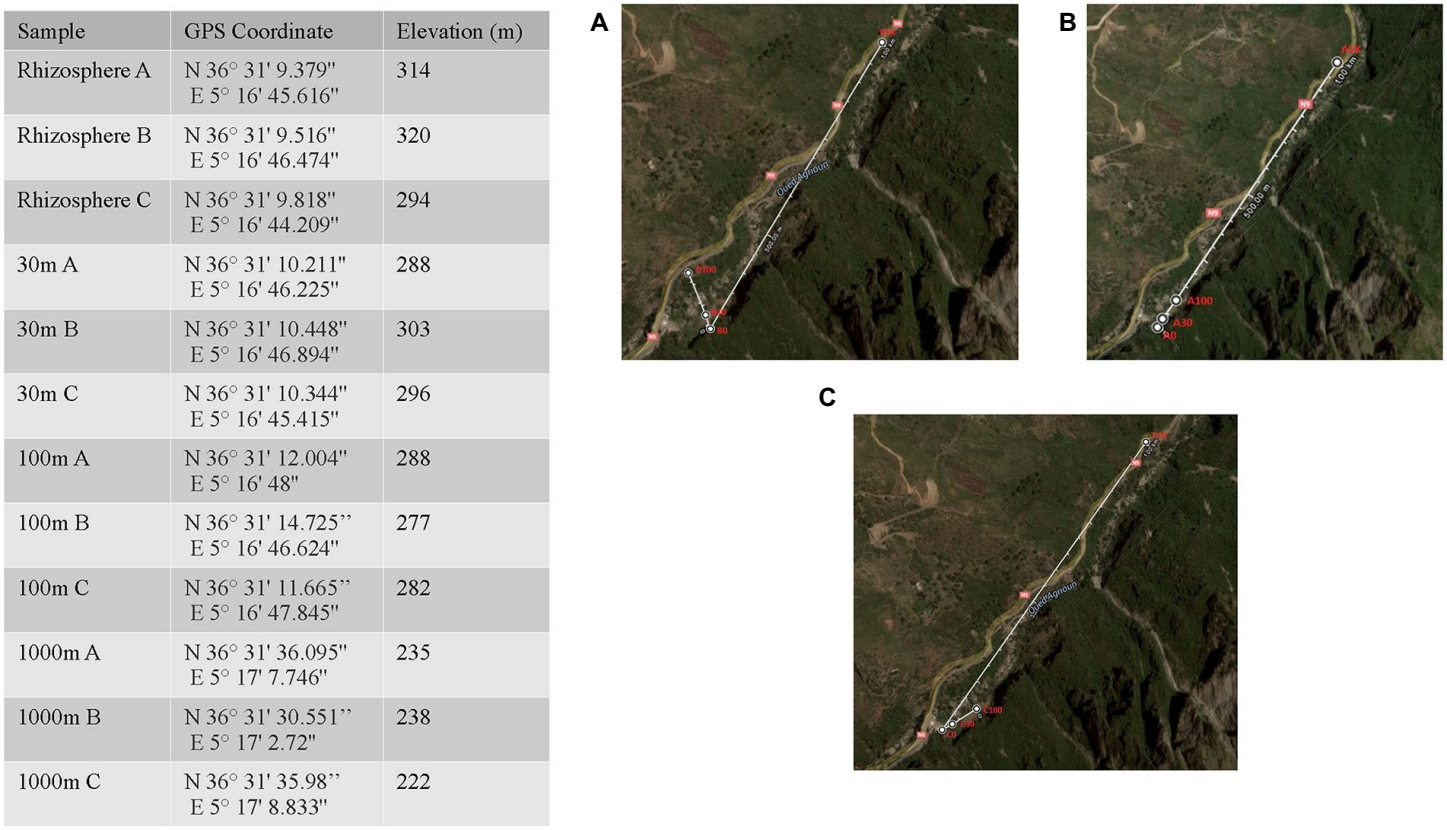
Location and geographic information for Algerian phytomicrobiome, soil, rhizosphere samples used in this study. Map insets **A–C** correspond to the three *Coriaria myrtifolia* plant replicates.

The sampling site, “Les Gorges de kharrata Bejaia,” is located in the Mediterranean low-altitude limestone cliffs in Northern Algeria characterized by Rendzina soil under sub-humid bioclimate (average annual rainfall is 881 mm; average annual temperature of 13.9°C, the absolute minimum and maximum temperatures are −9.30 and 41°C, respectively), which considered is appropriates for rupicolous vegetation (Bouchibane et al., 2021). Leaf, stem, fruit, root, and nodule tissues were sampled from three distinct 20 m tall *C. myrtifolia* plants (Figure 1) and placed in sterile plastic pouches. Latex gloves were worn by personnel and changed frequently during the sampling process. A subset of leaves, stems, fruits, roots, and nodules together with seeds of *C. myrtifolia* were washed with sterile distilled water and surface sterilized; by shaking in 30% (v/v) H_2_O_2_ for 30 min and then aseptically rinsed eight times with sterile water. This first subset of samples represents the endophytic community. A second subset of leaves, stems, fruits, roots, and nodules together with seeds of *C. myrtifolia* were washed with sterile distilled water several times and were used in the DNA extract process as non-disinfected plant tissues. The second subset of samples represents the epiphytic community. *Frankia* isolation from root nodules was performed as previously described by Gueddou et al. (2019). Soils (10 g) from the rhizosphere were sampled at a depth of 20 cm starting at the base of the same three *C. myrtifolia* plants and along 1 km linear series of plots extending from three *C. myrtifolia* stands. A subset of soils samples was used to inoculate axenic seedlings of *C. myrtifolia* grown on sterile sand and watered with BD medium (Broughton and Dilworth, 1971) without nitrogen source. One month-old seedlings (*n* = 10) were inoculated by adding 10 g of each soil sample. Fifteen non inoculated seedlings were used as negative control. Growth was maintained for 10 months, and roots were checked monthly for nodule formation.

Extraction of DNA from surface-sterilized and washed nondisinfected plant tissues was performed using Plant DNeasy kits (Qiagen, Hilden, Germany). For soil samples, the DneasyPowerSoil Kit (Qiagen, Valencia, CA, United States) was used. Extracted DNA was treated with 0.5 μl of RNase A (10 mg/ml) for 30 min at 37°C to remove RNA from the sample (Qiagen). For each sample, extractions were performed in triplicate. For the nodule samples, three well developed nodules were used per replicate sample.

### PCR amplification and paired-end sequencing of the V4 hypervariable region of the 16S rRNA gene amplificon

Prokaryotic community profiles from plant organs and soils were investigated using the V4 hypervariable region of the 16S subunit rRNA gene. The products were amplified and sequenced following the Earth Microbiome Project 515F/806R protocol (Thompson et al., 2017). Paired-end sequencing of the amplification products was performed using the Illumina HiSeq2500 platform (Illumina, San Diego, CA, United States) as previously described (Louati et al., 2020).

### Data processing and analysis of amplicon sequences

The 16S rRNA amplicon sequences of each sample were imported into the Quantitative Insights into Microbial Ecology (QIIME) 2 environment (version qiime2-2017.12) for processing and downstream analysis (Caporaso et al., 2010; Bolyen et al., 2019). Despite repeated sequencing and read pooling, poor quality at the 5’end of reverse reads resulted in decreased pairing and a significant loss of sequencing depth. Therefore, a single-end analysis using only the forward reads was selected. The forward reads were trimmed, and error corrected with DADA2 (Callahan et al., 2016) removing chimeric reads and truncating reads at 240 bp to exclude reads with a Phred quality below 35. Phylogenetic diversity of the resulting features was determined with Multiple Alignment using Fast Fourier Transformation (MAFFT; Katoh and Standley, 2013) and fasttree (Price et al., 2010) using the QIIME2 phylogeny plugin (Bokulich et al., 2018). Taxonomic assignment of the sequence variants in the feature table was accomplished using the QIIME2 feature-classifier plugin (Bokulich et al., 2018). A feature classifier was trained using Naive-Bayes (Pedregosa et al., 2011) and the full-length SILVA 99% identity 16S database (release 138; Quast et al., 2013). The resulting classifier was used with the classify-sklearn method (Pedregosa et al., 2011) to assign taxonomy to the feature table. Genus and phyla level data tables were exported from QIIME2 for diversity analysis in R. For the sake of continuity with previous amplicon-based microbial community studies, we will refer to sequence variants as operational taxonomic units (OTUs; Nguyen et al., 2016).

Alpha diversity in each of the four data sets was determined based on observed operational taxonomic units (OTUs) with taxonomies assigned at both the phylum and genus level. The Shannon Diversity Index (Shannon, 1948) was used to calculate alpha diversity of each sample. Python scripting was used to process and format QIIME2 feature table data which was plotted in R using ggplot2 (Wickham, 2016). Non-metric Multidimensional Scaling (NMDS) analysis (Minchin, 1987) was carried out in R using separate exported QIIME2 feature tables collapsed to genus level taxonomic OTUs. The metaMDS function in the vegan package of R (Dixon, 2003; Oksanen et al., 2018) was used for the NMDS analyses with Bray-Curtis distance (Sørensen, 1948), a trymax of 50 and K of 2. Before NDMS analysis, all feature counts in each sample were relativized by the total number of features in the sample to control for extraction and amplification error. The veganCovEllipse function (Torondel et al., 2016) was used to generate 95% confidence intervals within the ordination space. Ellipses were plotted to represent the confidence interval information and the NMDS data plotted in the sample figure. NDMS figures were generated with ggplot2 (Wickham, 2016) in R. Relative abundance taxonomic bar plots were generated with ggplot2 (Wickham, 2016) using QIIME2 feature tables with taxa collapsed to OTUs at the phylum and genus level.

After taxonomic assignment at the genus level (QIIME2 taxa L6) was completed in QIIME2, reads assigned to the genus *Frankia* in samples from the roots, rhizosphere, and all three bulk soil types were exported and reassigned to *Frankia* clade level bins with a custom python script. Specifically, a BLAST database was made using the v4 region of the 45 previously sequenced *Frankia* strains. The average expect value for alignments between each read and the all the *Frankia* strain v4 sequences in each clade was calculated. All reads were then assigned to a bin corresponding to the clade with the lowest average expect value for that read. The number of reads in each clade bin was normalized by total reads per sample and used to generate a heat map.

### Statistical analysis

For all statistical analyses, the significance of level was set to *p* < 0.05. R was used for all statistical analysis and visualization.^1^ Significant differences in alpha diversity were determined using ANOVA. Pairwise Permutational Multivariate ANOVA (PERMANOVA) analyses (Anderson, 2014) were carried out in R using the adonis function in the vegan package (Dixon, 2003; Oksanen et al., 2018) to test the significance of between group differences observed in the NMDS analyses. Bray-Curtis distance (Sørensen, 1948) and Bonferroni correction were used in each PERMANOVA analysis (Anderson, 2014). Simper analysis (Clarke, 1993) was carried out to assess the contribution individual features had on the observed differences between samples. Simper analyses (Clarke, 1993) were performed using the vegan package in R (Dixon, 2003; Oksanen et al., 2018) with the default parameters to gain insight into the taxa driving differences between groups observed in NMDS and PERMANOVA.

## Results

### Phytomicrobiomes of *Coriaria myrtifolia*

Table 1 summarizes the sequencing results for *C. myrtifolia* phytomicrobiome, rhizosphere, and surrounding associated soils. Rarefaction curves indicate that the sequencing depth was adequate to capture most of the observable diversity (Supplementary Figure S1).

**Table 1:** Summary of sequencing results for *C. myrtifolia* phytomicrobiome, rhizosphere and surrounding associated soils.

***Sample ID***	***Total reads***	***Average reads***	***Average OTUs/sample***
**All plant samples**	1,075,722.00	35,857.40	91.7
**Endophyte Samples**	664,416.00	44,294.40	71.6
Leaf	36,553.00	12,184.30	15.7
Fruit	24,452.00	8,150.70	56
Stem	60,669.00	20,223.00	29.67
Root	194,431.00	64,810.30	88.67
Nodule	348,311.00	116,103.70	168
**Epiphyte samples**	411,306.00	27,420.40	111.9
Leaf	54,075.00	18,025.00	12.3
Fruit	25,625.00	8,541.70	76.33
Stem	16,017.00	5,339.00	32.33
Root	190,677.00	63,559.00	133.67
Nodule	124,912.00	41,637.30	304.67
**Soil samples**	129,135.00	10,761.30	187.0
Rhizosphere	83,749.00	27,916.30	350.0
30 m away	18,181.00	6,060.30	168.0
100 m away	13,616.00	4,538.70	116.0
1,000 m away	13,589.00	4,529.70	114.0

**Figure 2:**
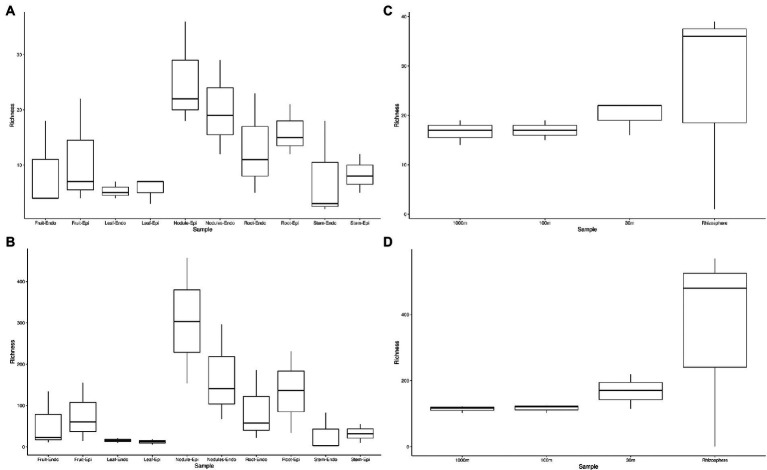
Phylum and genus level alpha diversity of *Coriaria myrtifolia* phytomicrobiome, soil, and rhizosphere microbiomes. **(A,B)** represent *C. myrtifolia* phytomicrobiome, while **(C,D)** represent *C. myrtifolia* soil and rhizosphere microbiomes. **A** and **C** are at the phylum-level, while panels show the genus-level diversity. Boxes represent 25 to 75th percentile variance around the mean. Whiskers represent 1.5 interval quartile ranges. Data points outside of the whiskers are considered outliers and are plotted separately.

**Figure 3:**
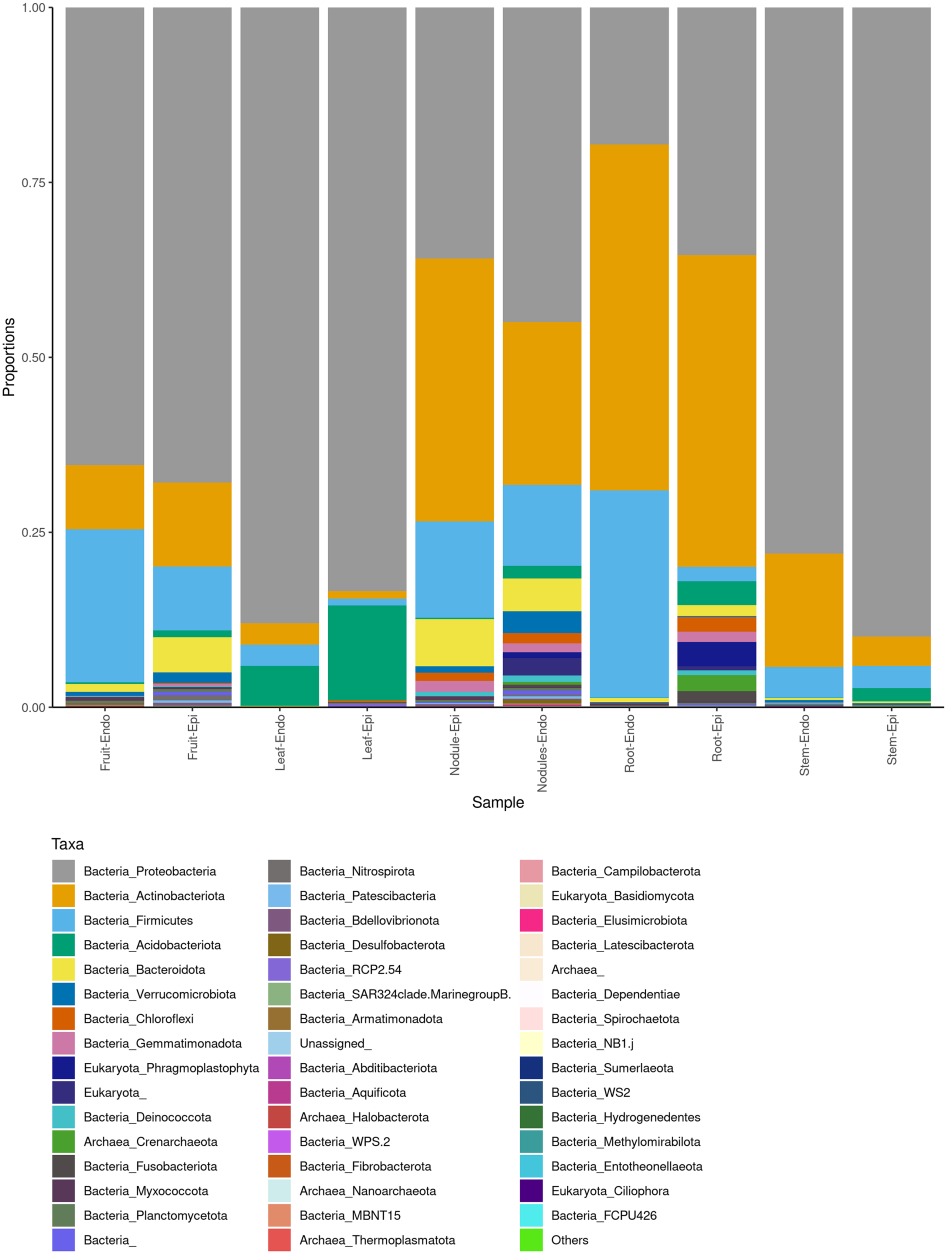
Phylum level taxonomy of *Coriaria myrtifolia* phytomicrobiome. Average 16S amplicon data show the relative abundance of each phylum after rarefying. Bars represent the average relative abundance of a given taxa across three replicate samples. Both epiphytic (Epi) and endophytic (Endo) communities are presented.

**Figure 4:**
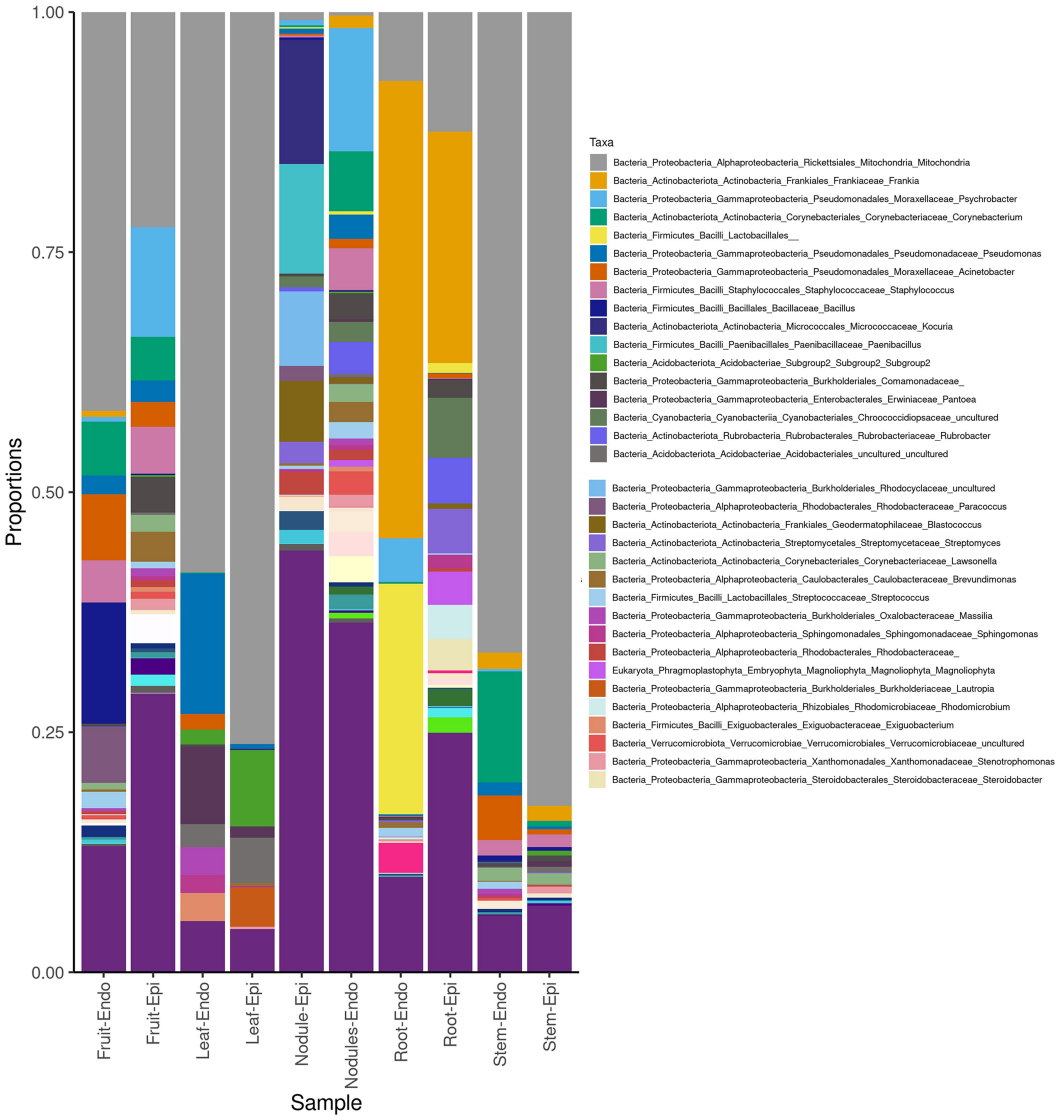
Genus-level taxonomy of *Coriaria myrtifolia* phyotmicrobiome. Average 16S amplicon data shows he relative abundance of each phylum after rarefying. Bars represent the average relative abundance of a given taxa across three replicate samples. Both epiphytic (Epi) and endophytic (Endo) communities are presented.

The measured Shannon diversity metric (Figures 2A,B) of epiphytic and endophytic communities among the various plant organs was not significantly different from each other (Student’s T-test, *p* > 0.05). Community richness was significantly higher in roots and nodules than for leaves, stems, and fruit (Figures 2A,B). Overall taxonomic compositions of the microbiomes for each plant organ at phylum-level are shown in Figure 3, while Figure 4 shows genus-level distribution. The phyla, *Cyanobacteria* and *Proteobacteria*, dominated the leaf and stem microbiome, while fruit contained an abundance of *Proteobacteria*, *Actinobacteria*, and *Cyanobacteria*, which were followed by Firmicutes and Bacteroidetes. Root and nodule communities contained *Actinobacteria*, *Proteobacteria*, *Firmicutes*, *Cyanobacteria*, *Bacteroidetes*, and other phyla. Interestingly at family level nodules contained *Nocaridiaceae, Phylobacteriaceae, Flammeovirgaceae, Erythrobctaeriaceae*, and *Rhodospirillaceae*, while in root we detected *Frankiaceae* and *Promicromonosporaceae*.

### Rhizosphere and outward soil plot microbiomes

**Figure 5:**
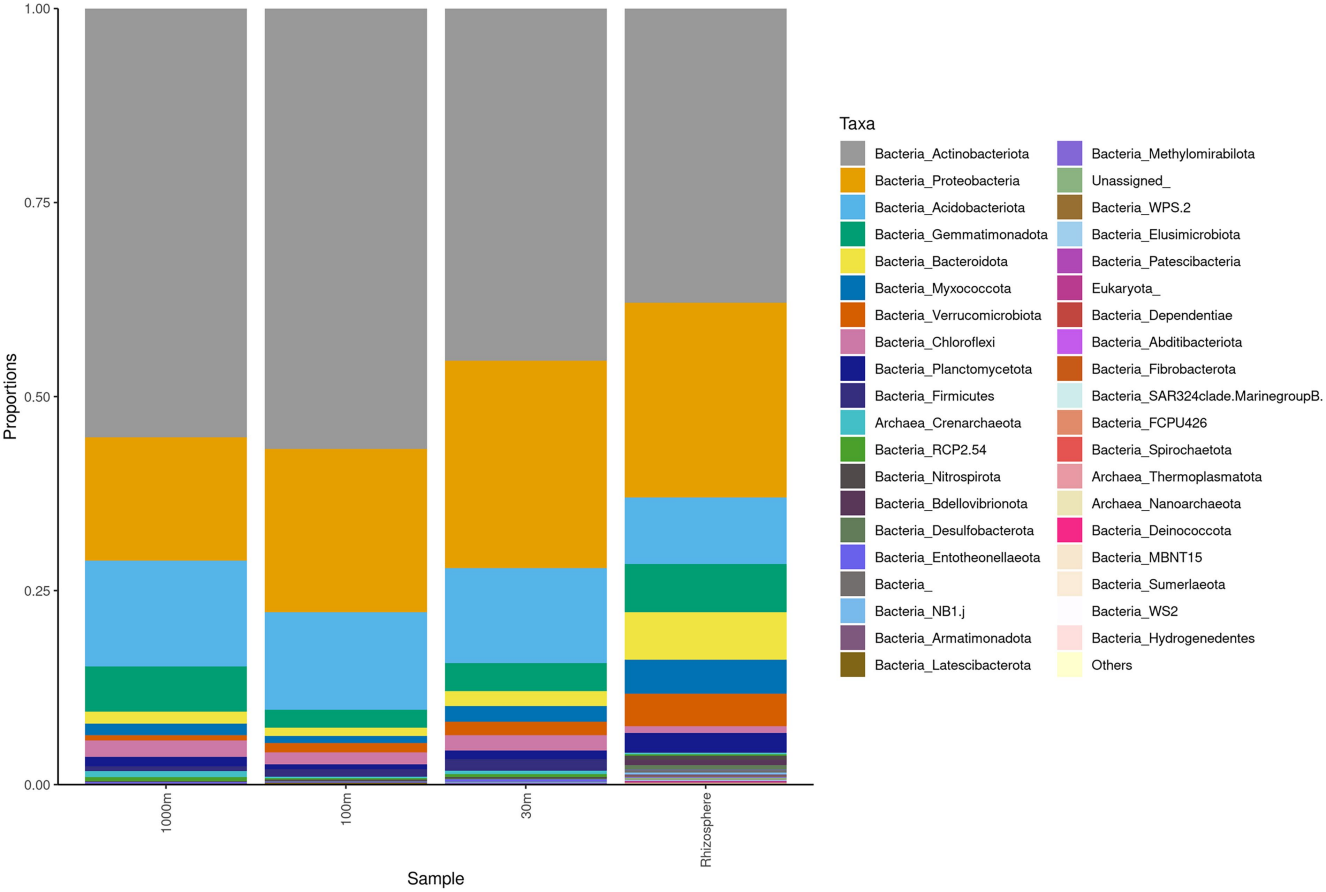
Phylum level taxonomy of *Coriaria myrtifolia* rhizosphere and nearby soil samples. Average 16S amplicon data shows the relative abundance of each phylum after rarefying. Bars represent the average relative abundance of a given taxa across three replicate samples.

Sequencing of the *C. myrtifolia* rhizosphere and nearby soil samples resulted in 129,135 total reads after quality filtering and 4,551 unique features in 12 samples. Features per sample ranged from 203 to 62,808. Table 1 summarizes these results. Rhizosphere soil has the highest number of average OTUs per sample. The remaining soil samples had lower average OTUs per sample but were similar among the different distances. Alpha diversity (Figures 2C,D) measured by the Shannon diversity metric confirmed that the rhizosphere was significantly different from the associated bulk soils (Student’s T- test; *p* > 0.05) Rarefaction curves indicate that sequencing depth of the soil and rhizosphere samples was adequate to capture most of the diversity present at the sites (Supplementary Figure S2). At phylum level, community structures for soil samples differed only in terms of taxon abundance (Figure 5) and were composed of *Proteobacteria* and *Actinobacteria* followed by *Acidobacteria*, *Gemmatimonadetes, Bacteriodetes*, *Verrucomicrobia*, *Planctomycetes*, and others. Supplementary Figure S3 shows a genus-level distribution.

### Beta diversity of phytomicrobiome

**Figure 6:**
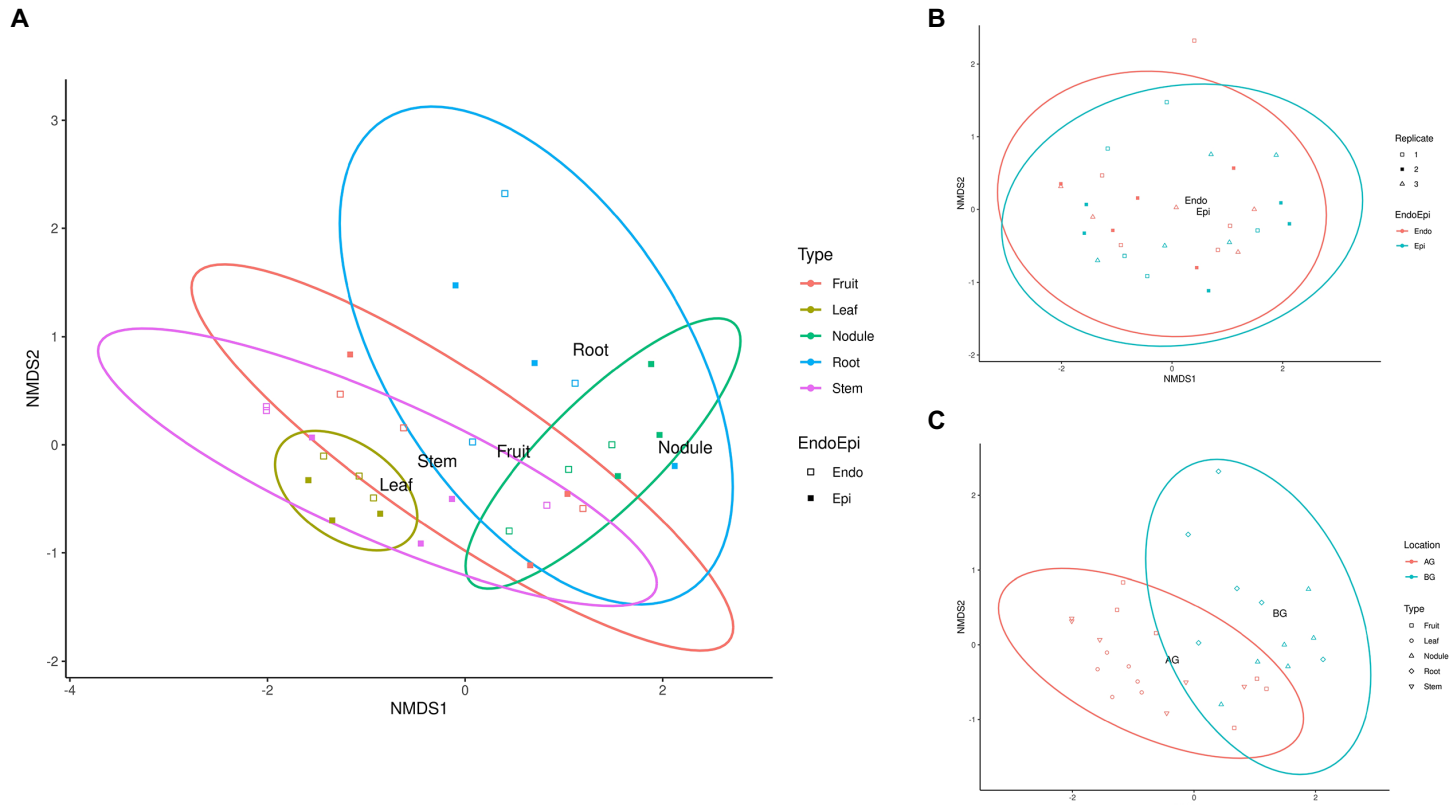
Non-metric Multidimensional Scaling (NMDS) ordination of *C. myrtifolia* phytomicrobiome. Ordinations of the phytomicrobiome communities were produced by the Bray-Curts distance metric. **(A)** shows NMDS for each plant tissue and both epiphytes and endophyte populations. Final ordination stress = 0.139. Procustes rnse <0.001, and max resid = 0.002. Point and ellipse color correspond to tissue type and point fill corresponds to endophyte (unfilled) and epiphyte (filled) samples. **(B)** shows NMDS for the epiphyte and endophyte communities. Final ordination stress = 0.139, Procrustes rmse <0.001, and max resid = 0.002. Point and ellipse color correspond to epiphyte (blue) and endophyte (red) samples. Point shape and fill correspond to sample replicate. **(C)** shows NMDS for the above- and below-ground phytomicrobiome communities. Final ordination stress = 0.139, Procrustes rmse <0.001, and max resid = 0.002. Point and ellipse color correspond to above ground samples (AG: fruit, leaf, and stem), and below ground samples (BG: root and nodule). Point shape corresponds to sample types with endophyte and epiphyte samples combined.

**Figure 7:**
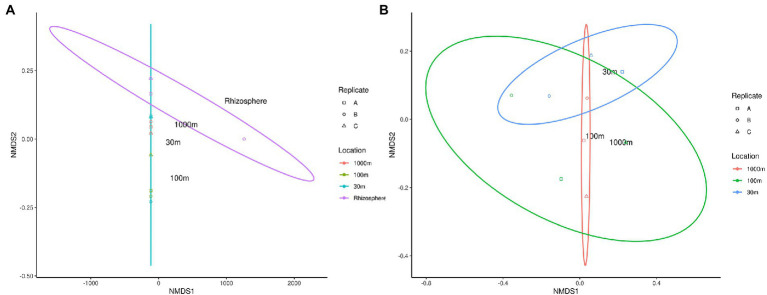
NMDS ordination of *C. myrtifolia* rhizosphere and nearby soil samples. Ordinations of the microbiome communities were produced by the Bray-Curts distance metric. **(A)** shows NMDS for the results for *C. myrtifolia* rhizosphere and nearby soil samples. Final ordination stress—0.107, Procrustes rnse, 0.002, and max resid—0.004. Data point colors correspond to distance from the *C. myrtifolia* plant. **(B)** shows the results with only the bulk soil samples and the rhizosphere removed to emphasize differences among those samples. Final stress = 0.130, Procrustes rmse < 0.001, max resid < 0.001. Data point colors correspond to distance from the *C. myrtifolia* plant. Data point shape corresponds to sample replicates.

Beta diversity of phytomicrobiome of the phytobiomes was analyzed to resolve how the samples differed by tissue type, lifestyle (endophyte or epiphyte), or location (above- or below-ground level). The Bray–Curtis distance metric was determined for the samples and ordinated through NMDS analysis. Figure 6 shows the results of this analysis. The tissue clustered together (Figure 6A) and had significant differences between below-ground samples (roots and nodules) and above-ground samples (fruit, leaves, and stems; Figure 6C). However, no difference was identified between endophyte and epihyte samples (Figure 6B). PERMANOVA analysis was performed to determine whether there are significant differences in community structures among the samples (Supplementary Tables S1–S4). Roots were significantly distinct from all other tissue groups individually except nodules. Nodules were significantly distinct from stem and leaf samples, but not from root samples. Fruit and leaf samples were also significantly distinct groups within the ordination. All sample types except stems were individually distinct from the sample types collectively. The NMDS (Figure 7) and PERMANOVA (Supplementary Table S4) analyses of the rhizosphere and associated soil samples from Algeria indicate the only significantly distinct sample type was the rhizosphere.

Simper analysis showed that *Proteobacteria, Firmicutes, Acidobacteria, Actinobacteria,* and *Bacteroidetes* were the most differentiating phyla level taxa between *C. myrtifolia* fruit and leaf tissue communities. Fruit and root communities, fruit and stem communities, leaf and nodule communities, leaf and stem communities, and nodule and root communities primarily differentiated by the same taxa: *Proteobacteria, Firmicutes, Acidobacteria, Actinobacteria,* and *Bacteroidetes*. For between the fruit and nodules communities as well as the nodule and stem communities *Proteobacteria, Actinobacteria, Firmicutes, Bacteroidetes*, and *Verromicrobia* were the most differentiating taxa. Leaf and root communities in addition to root and stem communities were differentiated most by *Proteobacteria, Firmicutes, Acidobacteria, Actinobacteria*, and *Phragmoplastophyta*. Simpler analysis of the *C. myrtifolia* phytomicrobiome at the genus level showed that *Frankia* plays a substantial role in differentiating the phytomicrobiomes of specific plant tissues despite being in a low abundance taxon. The genus *Frankia* was one of the top two most significant taxa characterizing the differences between fruit and nodule, leaf and root, nodule and root, and root and stem communities.

*Actinobacteria, Proteobacteria, Acidobacteria*, *Gemmatimonadota*, and *Bacteroidetes* were identified by Simper analysis as the most significant phyla contributing to the community differences found between and among all rhizosphere and soil samples from Algeria.

### Detection of the different *Frankia* clusters

**Figure 8:**
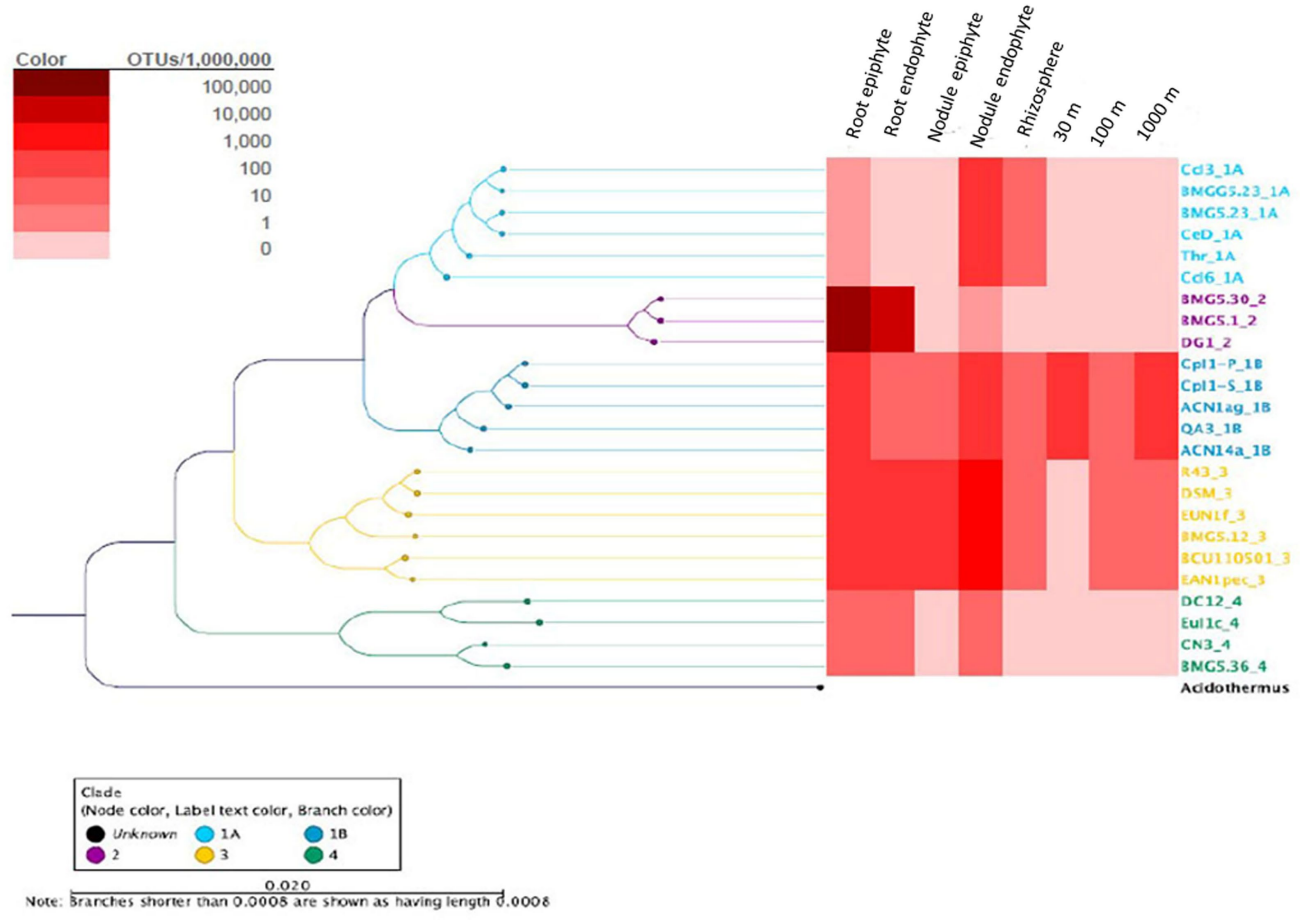
Remapped *Frankia* genus reads to clade level phylogeny. *Coriaria myrtifolia* rhizosphere, root, nodule, and nearby soil samples are shown. Endophyte and epiphyte communities of the nodule and root samples are shown. Rhizosphere and bulk soil samples and distance from the plant are also presented. Heat-map colors correspond to reads per million for a given set of samples.

Further analysis of the *Frankia* OTUs by clade level binning (Figure 8) detected *Frankia* cluster 2 in both endophyte and epiphyte locations in the root and nodule samples, while cluster 1a was detected in root, nodule, rhizosphere, and outward soil samples. Cluster 3 was present in all samples excepting 30 m soil sample. Cluster 1c occurred in root, nodule, and rhizosphere, while cluster 4 was detected in nodule and root. To confirm these results, a plant trapping assay was performed. The mean number of nodules formed on *C. myrtifolia* seedlings ranged from 5 for rhizospeheric, 2 for 30 m and two nodules for each of 100 m, and 1 km soil samples. Time courses of nodulation were shorter for rhizospheric soil (3 months) than other soil samples (6 months).

## Discussion

### *Coriaria myrtifolia* phytomicrobiome

As expected, sequences are mostly assigned to the Bacteria domain while Archaea are not frequently detected (Müller et al., 2016). Overall, the *C. myrtifolia* phytosphere is composed of *Proteobacteria*, *Actinobacteria*, Cyanobacteria, and *Firmicutes* as the most abundant phyla. Core microbiomes of several plant species including crops, cultivated, wild, and trees include abundant *Proteobacteria*, *Actinobacteria*, and *Bacteroidetes* together with a slighter fraction of *Firmicutes* (Bulgarelli et al., 2013; Bogas et al., 2015; Müller et al., 2016). The prevalence of bacterial phyla composition varied between above- and below-ground and to lesser extent among plant organs. This result has been attributed to the selective gradient in the soil from the roots to aerial part of plant tissues (Müller et al., 2016). Although epiphyte tissue samples had higher numbers of OTUs than endophytes samples, our results showed that most organ epiphyte and endophyte communities were not significantly different. Several studies reported that epiphytes versus endophytes differ in term of variation in taxon relative abundances (Müller et al., 2016) where epiphytic bacteria are usually more abundant than those of endophytes (James et al., 2002; Wallace et al., 2018). *Cyanobacteria* were most abundant and diverse at every taxonomic rank in leaf, stem, and fruit. This phylum was also described to predominate in fruit, leaf, flower, and stem of Jingbai Pear trees (Ren et al., 2019), rhizoplane of *Spartina alterniflora* (Hong et al., 2015), and bark, stem, needle, and root tissues of spruce (Ren et al., 2019), while others have reported that *Cyanobacteria* are considered absent or infrequently occurring in leaves (Redford et al., 2010; Rigonato et al., 2016). These photosynthetic and for some of them diazotrophic bacteria are wide-spread and aid the health and growth of many plant species (Stanier and Cohen-Bazire, 1977; Ringelberg et al., 2012). *Proteobacteria* and *Actinobacteria* were present in leaves, stems, and fruits at lower abundance, which is coherent with the results for several plant species. Previous studies have shown that *Alpha*- and *Gamma*-*proteobacteria* generally predominate other bacterial groups in leaves with variable levels of *Beta-proteobacteria, Bacteroidetes, Firmicutes*, and *Actinobacteria* presence depending on plant species (Idris et al., 2004; Delmotte et al., 2009; Redford et al., 2010). Similar to *Cyanobacteria*, *Bacteroidetes* usually are not detected or detected at low levels in leaves (Lambais et al., 2006; Redford et al., 2010). In this study, *Bacteroidetes* were not present except in some fruit and nodule samples. *Bacteroidetes* are ubiquitous colonizers of all types of habitats on Earth (Thomas et al., 2011) producing diverse carbohydrate-active enzymes with large spectrum of substrates from plant, algal, and animal origin that are important in gut-microbiomes (Whitman et al., 1998). The diverse *Pedobacter* species (*Bacteroidetes*) are detected in *A. glutinosa* decaying nodules emphasizing their implication in decomposing process (Mcewan et al., 2017).

### Rhizocompartment microbiomes

Root microbiome of *C. myrtifolia* contained *Actinobacteria, Proteobacteria, Firmicutes, Cyanobacteria, Bacteroidetes*, and others, which is very similar to composition of *Alnus glutinosa* root microbiome (Thiem et al., 2018), *Casuarina glauca* (Ghodhbane-Gtari et al., 2021), several Legume roots (Miranda-Sánchez et al., 2016; Xiao et al., 2017; Zgadzaj et al., 2016), and rice (Edwards et al., 2015; Roman-Reyna et al., 2020).

It is commonly accepted that actinorhizal nodules are a special niche for symbiotic nitrogen fixation, which is primarily colonized by *Frankia*. However, as it has been shown by the culture-dependent and -independent methods, several non-*Frankia* coexist with *Frankia* (Ghodhbane-Gtari et al., 2010b, 2019, 2021; Ghodhbane-Gtari and Tisa, 2014). At phylum level, the *C. myrtifolia* nodule microbiome differs from root only in term of relative abundance of each phylum and being composed of *Bacteroidetes, Proteobacteria*, and *Actinobacteria*. Similar results were shown for *A. glutinosa* nodule microbiome (Mcewan et al., 2017). At genus rank, *Flavobacteria, Nocardia*, *Rhodococcus*, *Psychrobacter*, and *Corynebacteria* are the most predominant together with *Phyllobacter*, *Erythrobacter*, *Erythrobacter*, and *Flammeovirgacia* genera.

Interestingly, the structure of rhizosphere microbiome (Figure 5) at phylum level is similar to root and nodule microbiomes (Figure 3). The root bacterial assemblage is derived mostly from rhizosphere and is established rapidly within a few days after seed germination and subsequently shaped by root exudates and plant genotype (Edwards et al., 2015; Hacquard et al., 2015). This filtration phenomenon is related to the roots affecting the nutrient supply and the physicochemical features of the rhizosphere as well as changes in the environment from soil to endosphere (Gottel et al., 2011; Edwards et al., 2015). Filtration effects were also proposed for legume rhizocompartments through spatial gradients in the following order: nodule > root > rhizosphere > root (Miranda-Sánchez et al., 2016; Xiao et al., 2017).

### *Frankia* cluster 2 dispersal outward from the *Coriaria myrtifolia* rhizosphere

While the presence of *Frankia* cluster 2 (the host compatible microsymbionts) and cluster 4 (asymbiotic *Frankia* that are often isolated from *Coriaria* nodules; Mirza et al., 1992; Gueddou et al., 2017) was expected, the occurrence of cluster 1 and the super-abundance of cluster 3 in *C. myrtifolia* nodule, root, and rhizosphere was surprising. As previously stated, clusters 1 and 3 are known to ubiquitously distributed in soils independent to the presence of compatible host-plants (Paschke and Dawson, 1992; Nalin et al., 1997; Gtari et al., 2004). Cluster 4 is also known to be found free-living in soil (Wolters et al., 1997). The presence of cluster 1 was shown to be abundant in *Betula pendula* rhizosphere (Smolander, 1990). The occurrence of cluster 2 in the *A. glutinosa* rhizosphere has been demonstrated (Nouioui et al., 2013). Cluster 3 strains have been isolated from the incompatible host *Casuarina* nodule (Nazaret et al., 1989). Several *Frankia* cluster 3 strains have been isolated from *Ceanothu*s spp. which like *Coriari*a spp. is typically infected by *Frankia* cluster 2 strains (Lechevalier and Ruan, 1984; Lechevalier, 1985; Baker, 1987). Using a glnA sequencing approach, *Frankia* cluster 3 strains were identified in *Ceanothus* nodules found in New England (Ktari et al., 2017). *Frankia* cluster 3 strains were isolated from *Coriaria* nodules obtained from Algeria but unfortunately, these isolates were lost (M. Gtari unpublished data). Furthermore, *Frankia* clusters 1 and 3 strains have been shown to dominate or co-dominate in the incompatible host rhizosphere (Samant et al., 2016; Rodriguez et al., 2016). Similar results have been shown for the presence of rhizobia as endophytes in the nodules of legumes other than their hosts (Gage, 2004; Peix et al., 2015; Xiao et al., 2017).

Blastmapping sub-genus level analysis of the metagenome results from 30 and 100 m and 1 km soil samples extending from *C. myrtifolia* rhizospheres failed to detect cluster 2 contrary to plant trapping bioassay where all seedlings were nodulated. Analysis of host rhizosphere microbiome showed that cluster 2 occupied only a tiny fraction of the microbiome (Battenberg et al., 2017). A quantitative PCR approach indicated that members of cluster 2 might not be present in significant amounts in all soils (Ben Tekaya et al., 2017). Similarly, the microsymbionts of soybean and alfalfa, *Ensifer* and *Bradyrhizobium* respectively, were relatively rare in bulk soil (Xiao et al., 2017). This situation was also shown for compatible rhizobial species for *Phaseolus vulgaris* that is a member of “rare rhizobial biosphere” in the bulk soil but is strongly found enriched on root surfaces and in nodule habitats (Miranda-Sánchez et al., 2016).

## Conclusion

Neurotoxicity and wild nature of *C. myrtifolia* did not greatly shape the phytomicrobiome as it is comparable to several wild or cultivated, herbaceous, or woody plants. The filtration effects of the rhizocompartments (rhizosphere, root, and nodule) was observable in the actinorhizal species *C. myrtifolia*. These rhizocompartiments are characterized by the occurrence of members of all four *Frankia* clusters. This fact could be possibly linked to the need of *C. myrtifolia* to a wider diversity of *Frankia* microsymbionts with diverse growth-promoting potentialities to cope with the harsher challenges of its wild status. Another possible explanation is that the actinorhizal rhizosphere may also be a primary refuge for the proliferation of all *Frankia* clusters. Further analysis on other actinorhizal species should clarify this phenomenon. Compared to other *Frankia* clusters, cluster 2 infective units are present in a less extended amount even in the presence of compatible host species. This amount will further drop outward the rhizosphere of its host plants.

## Data availability statement

The original contributions presented in the study are publicly available. This data can be found here: NCBI, PRJNA553505, and PRJNA553534.

## Author contributions

MG, FG-G, HC-S, and LT conceived the study. AK, IS, and ES performed the research. ES, FG-G, HC-S, LT, and MG analyzed the data. MG, FG-G, ES, and LT wrote the manuscript. All authors contributed to the article and approved the submitted version.

## Funding

This material is based upon work supported by the New Hampshire Agricultural Experiment Station, through joint funding of the National Institute of Food and Agriculture, U.S. Department of Agriculture, and the state of New Hampshire. This is Scientific Contribution Number 2947. This project (LST) was supported by the USDA National Institute of Food and Agriculture Hatch 1019869 (LST), the College of Life Science and Agriculture at the University of New Hampshire-Durham, and the Ministère de l’Enseignement Supérieur et de la Recherche Scientifique, Tunisia USCR Bactériologie Moléculaire & Génomique, INSAT-Université de Carthage.

## Conflict of interest

The authors declare that the research was conducted in the absence of any commercial or financial relationships that could be construed as a potential conflict of interest.

## Publisher’s note

All claims expressed in this article are solely those of the authors and do not necessarily represent those of their affiliated organizations, or those of the publisher, the editors and the reviewers. Any product that may be evaluated in this article, or claim that may be made by its manufacturer, is not guaranteed or endorsed by the publisher.

## Supplementary material

The Supplementary material for this article can be found online at: https://www.frontiersin.org/articles/10.3389/fmicb.2022.1027317/full#supplementary-material

## Footnotes

^1^ http://www.R-project.org/
