# Supplementary material for: Decrypting phytomicrobiome of the neurotoxic actinorhizal species, Coriaria myrtifolia, and dispersal boundary of Frankia cluster 2 in soil outward compatible host rhizosphere
Source: Front Microbiol. 2022 Nov 10;13:1027317. doi: 10.3389/fmicb.2022.1027317 (PMC9684332; doi:10.3389/fmicb.2022.1027317)
Supplement: Supplementary file 1 [file Data_Sheet_1.PDF]

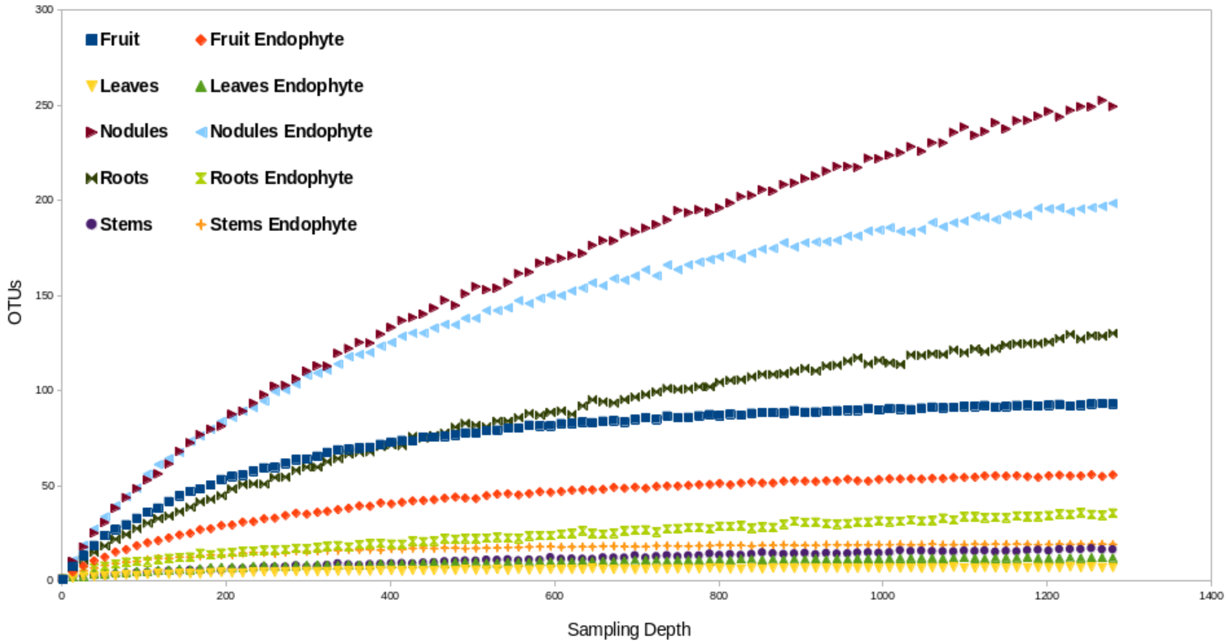

Figure S1. Average rarefaction plot of *C. myrtifolia* phtyomicrobiome samples

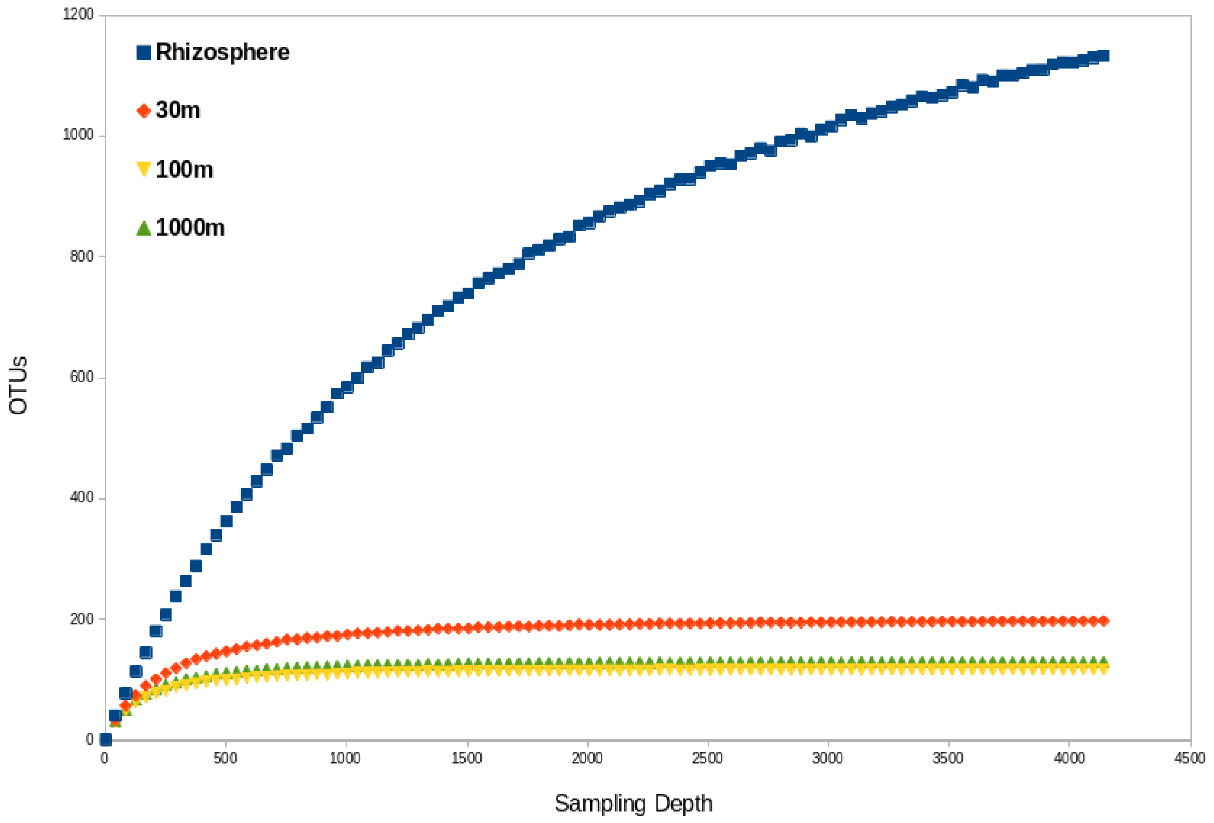

Figure S2. Average rarefaction plot of *C. myrtifolia* rhizosphere and nearby soil samples**.**

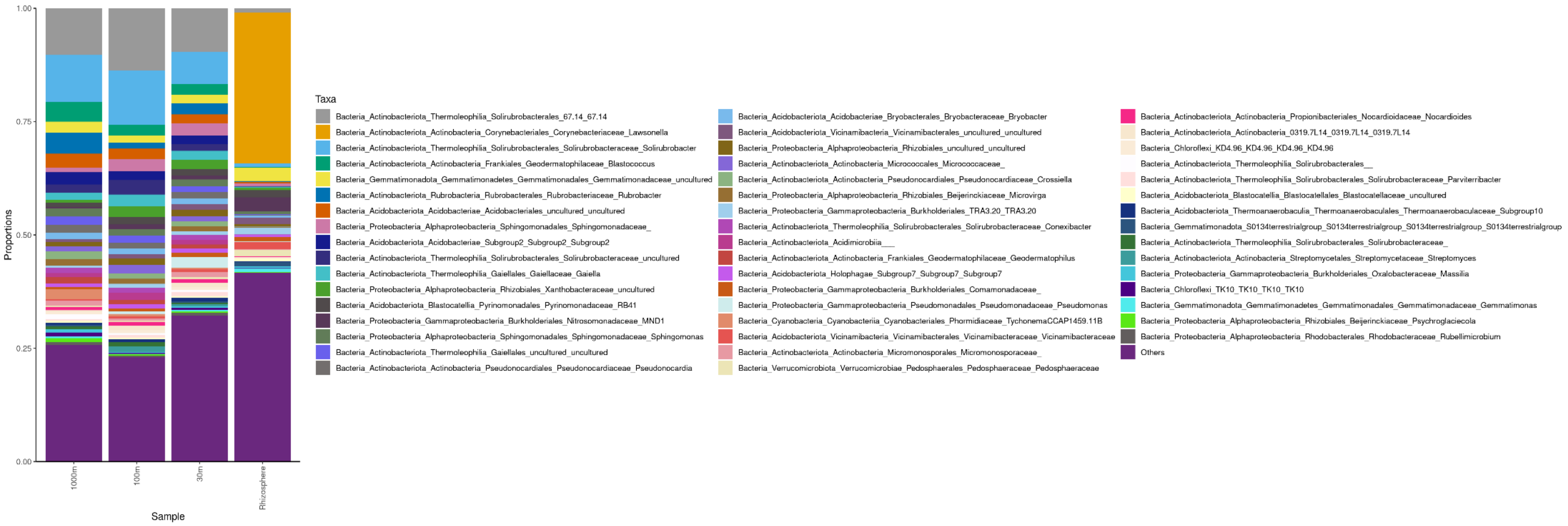

Figure S3. Genus-level taxonomy of *C. myrtifolia* rhizosphere and nearby soil samples. Average 16S amplicon data shows he relative abundance of each phylum after rarefying. Bars represent the average relative abundance of a given taxa across three replicate samples.

Table S1. Phylum level PERMANOVA analysis results for the *C. myrtifolia* phytomicrobiome. PERMANOVA scores were calculated using the adonis R function using Bray-Curtis distance and a phylum level feature table generated using QIIME2. PERMANOVA comparisons were made for each pair-wise tissue type combination.

Comparison	***F Model***	***R2***	***P Value***
Fruit vs. Stem	1.714	0.146	0.147
Fruit vs. Leaf	9.646	0.491	0.008
Fruit vs. Root	3.280	0.247	0.044
Fruit vs. Nodule	3.980	0.285	0.026
Stem vs. Leaf	1.734	0.133	0.133
Stem vs. Root	2.579	0.205	0.047
Stem vs. Nodule	5.444	0.353	0.002
Leaf vs. Root	6.576	0.397	0.004
Leaf vs. Nodule	13.694	0.578	0.004
Nodule vs. Root	1.342	0.118	0.191

Table S2. Phylum-level PERMANOVA analysis results for the microbiome of the *C. myrtifolia* rhizosphere and nearby soil. PERMANOVA scores were calculated using the adonis R function using Bray-Curtis distance and a phylum level feature table generated using QIIME2. PERMANOVA comparisons were made for each pair-wise tissue type combination.

Comparison	*F Model*	*R2*	*P Value*
Rhizosphere vs. All	7.598	0.432	0.005
30 m vs. All 0.657	0.657	0.062	0.706
100 m vs. All	0.904	0.083	0.470
1000 m vs. All	1.382	0.121	0.160
Rhizosphere vs. 100 m	3.466	0.464	0.100
Rhizosphere vs. 1000 m	4.258	0.516	0.100
Rhizosphere vs. 30 m	2.901	0.420	0.100
100 m vs. 1000 m	2.011	0.335	0.100
100 m vs. 30 m	0.651	0.140	0.900
1000 m vs. 30 m	1.886	0.320	0.100

Table S3. Genus-level PERMANOVA analysis results for the *C. myrtifolia* phytomicrobiome. PERMANOVA scores were calculated using the adonis R function using Bray-Curtis distance and a genus-level feature table generated using QIIME2. PERMANOVA comparisons were made for each pair-wise tissue type combination and between each tissue compared to all other tissue types collectively. AG =above-ground tissue (Fruit, Leaf, Stem), BG = below-ground tissue (root and nodule)

Comparison	***F Model***	***R2***	***P Value***
Fruit vs. Stem	1.207	0.108	0.280
Fruit vs. Leaf	4.637	0.317	0.003
Fruit vs. Root	2.473	0.198	0.015
Fruit vs. Nodule	2.007	0.167	0.053
Stem vs. Leaf	3.564	0.263	0.016
Stem vs. Root	3.148	0.239	0.005
Stem vs. Nodule	3.676	0.269	0.002
Leaf vs. Root	7.646	0.433	0.005
Leaf vs. Nodule	8.344	0.455	0.003
Nodule vs. Root	1.862	0.157	0.050
Fruit vs. All	1.473	0.050	0.150
Leaf vs. All	3.410	0.109	0.005
Nodule vs. All	4.834	0.147	0.002
Root vs. All	2.171	0.072	0.046
Stem vs. All	1.193	0.040	0.257
AG vs. BG	5.543	0.165	0.002

Table S4. Genus-level PERMANOVA analysis results for the microbiome of the *C. myrtifolia* rhizosphere and nearby soil. PERMANOVA scores were calculated using the adonis R function using Bray-Curtis distance and a genus-level feature table generated using QIIME2. PERMANOVA comparisons were made for each pair-wise sample type combination and each sample type vs. all other sample types.

Comparison	*F Model*	*R2*	*P Value*
Rhizosphere vs. All	7.598	0.432	0.005
30 m vs. All 0.657	0.657	0.062	0.706
100 m vs. All	0.904	0.083	0.470
1000 m vs. All	1.382	0.121	0.160
Rhizosphere vs. 100 m	3.466	0.464	0.100
Rhizosphere vs. 1000 m	4.258	0.516	0.100
Rhizosphere vs. 30 m	2.901	0.420	0.100
100 m vs. 1000 m	2.011	0.335	0.100
100 m vs. 30 m	0.651	0.140	0.900
1000 m vs. 30 m	1.886	0.320	0.100
